# 

**Published:** 1893-06-17

**Authors:** 


No. 351, Vol. XIV.—June 17th, 1893.

[Registered at the General Post Office as a Newspaper for transmission in the United Kingdom and Abroad.]

WITH EXTRA NURSING SUPPLEMEN[illegible]

Per Annum, 8s. 6d., post free.

Monthly Parts, 6d.; post free, 8d.

Ditto per Annum; 8s., post free.

### THE HOSPITAL

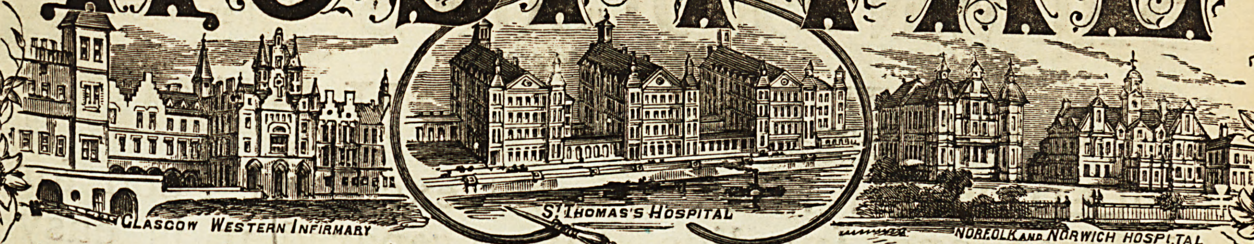

WITH EXTRA NURSING SUPPLEMENT.

No. 351. Vol. XIV.] SATURDAY, JUNE 17TH, 1893. [TWOPENCE.

